# Yeast model analysis of novel polymerase gamma variants found in patients with autosomal recessive mitochondrial disease

**DOI:** 10.1007/s00439-015-1578-x

**Published:** 2015-06-16

**Authors:** Magdalena Kaliszewska, Jakub Kruszewski, Biruta Kierdaszuk, Anna Kostera-Pruszczyk, Monika Nojszewska, Anna Łusakowska, Joel Vizueta, Dorota Sabat, Dorota Lutyk, Michał Lower, Dorota Piekutowska-Abramczuk, Aneta Kaniak-Golik, Ewa Pronicka, Anna Kamińska, Ewa Bartnik, Paweł Golik, Katarzyna Tońska

**Affiliations:** Faculty of Biology, Institute of Genetics and Biotechnology, University of Warsaw, Pawinskiego 5a, 02-106 Warsaw, Poland; Department of Neurology, Medical University of Warsaw, Banacha 1a, 02-097 Warsaw, Poland; Department of Virology, Faculty of Biology, University of Warsaw, Miecznikowa 1, 02-096 Warsaw, Poland; Department of Medical Genetics, Children’s Memorial Health Institute, Al. Dzieci Polskich 20, 04-730 Warsaw, Poland; Institute of Biochemistry and Biophysics, Polish Academy of Sciences, Pawinskiego 5a, 02-106 Warsaw, Poland; Department of Pediatrics, Nutrition and Metabolic Diseases, Children’s Memorial Health Institute, Al. Dzieci Polskich 20, 04-730 Warsaw, Poland

## Abstract

Replication of the mitochondrial genome depends on the single DNA polymerase (pol gamma). Mutations in the *POLG* gene, encoding the catalytic subunit of the human polymerase gamma, have been linked to a wide variety of mitochondrial disorders that show remarkable heterogeneity, with more than 200 sequence variants, often very rare, found in patients. The pathogenicity and dominance status of many such mutations remain, however, unclear. Remarkable structural and functional conservation of human POLG and its *S. cerevisiae* ortholog (Mip1p) led to the development of many successful yeast models, enabling to study the phenotype of putative pathogenic mutations. In a group of patients with suspicion of mitochondrial pathology, we identified five novel POLG sequence variants, four of which (p.Arg869Ter, p.Gln968Glu, p.Thr1053Argfs*6, and p.Val1106Ala), together with one previously known but uncharacterised variant (p.Arg309Cys), were amenable to modelling in yeast. Familial analysis indicated causal relationship of these variants with disease, consistent with autosomal recessive inheritance. To investigate the effect of these sequence changes on mtDNA replication, we obtained the corresponding yeast *mip1* alleles (Arg265Cys, Arg672Ter, Arg770Glu, Thr809Ter, and Val863Ala, respectively) and tested their effect on mitochondrial genome stability and replication fidelity. For three of them (Arg265Cys, Arg672Ter, and Thr809Ter), we observed a strong, partially dominant phenotype of a complete loss of functional mtDNA, whereas the remaining two led to partial mtDNA depletion and significant increase in point mutation frequencies. These results show good correlation with the severity of symptoms observed in patients and allow to establish these variants as pathogenic mutations.

**Electronic supplementary material:**

The online version of this article (doi:10.1007/s00439-015-1578-x) contains supplementary material, which is available to authorized users.

## Introduction

Mitochondria arose from an α-proteobacterial symbiont in an endosymbiosis event approximately 1.5 billion of years ago (Dyall et al. 2004; Gray et al. 1999). These organelles are present in all aerobic eukaryotic cells, and are crucial for energy production, and many other processes, like Fe-S cluster synthesis, Ca^2+^ homeostasis, fatty acid oxidation and apoptosis (Aon et al. 2014; Kasahara and Scorrano 2014; Stehling et al. 2014).

As a legacy of their endosymbiotic origin, mitochondria are the only organelles in animal and fungal cells containing their own DNA (mtDNA). Unlike nuclear DNA (nDNA), there are hundreds or even thousands of copies of mtDNA in a single cell. Usually all the copies of mitochondrial DNA are identical (homoplasmy), but mitochondrial DNA molecules with different sequence variants (polymorphic or pathogenic) can coexist with wild-type mtDNA (heteroplasmy) (Aanen et al. 2014).

Human mitochondrial DNA is a circular double-stranded molecule encoding 13 proteins which are subunits of the respiratory chain complexes, 22 tRNAs and 2 rRNAs (Holt et al. 2014; Mishra and Chan 2014). Therefore, the vast majority of 1000–1500 proteins necessary for the proper functioning of the organelle and cellular regulation of mtDNA integrity and copy number are encoded by nDNA and transported into the mitochondrion. The reciprocal influence of nuclear gene expression and processes located in mitochondria is referred to as intergenomic communication (Spinazzola and Zeviani 2007).

Mitochondrial diseases are a group of heterogenic multiorgan disorders, particularly affecting tissues with high energy requirement, like muscles and nerves. They may be caused by mtDNA mutations, nuclear gene mutations (for example in genes encoding subunits of respiratory chain complexes), or by mutations in nuclear genes encoding proteins involved in mtDNA replication and stability, having a secondary effect on mitochondrial DNA amount and quality (Spinazzola and Zeviani 2007).

Replication of human mitochondrial DNA depends entirely on nuclear-encoded proteins. The only DNA polymerase present in human mitochondria is polymerase γ, a member of the type A family of DNA polymerases. It functions as a heterotrimer and consists of a catalytic subunit (POLG) encoded by the *POLG* gene (15q26.1), and two accessory subunits (POLG2) encoded by the *POLG2* (17q23.3) gene. POLG is a 140 kDa protein composed of 1239 aa (the N-terminal 25 aa form the mitochondrial import signal and are cut off during import) and is capable of replicating DNA on its own; the interaction with POLG2 increases polymerase fidelity and processivity. Three domains can be distinguished in the catalytic subunit: the exonuclease domain with the proofreading activity (26–418 aa), the polymerase domain (756–1239 aa), and the linker region (419–755 aa) located between the two domains which is a platform for direct interaction with one of the accessory subunits (Graziewicz et al. 2006; Longley et al. 2005; Stumpf and Copeland 2011). An alternative localization of the POLG domain boundaries was proposed after the publication of the crystal structure of the Pol γ holoenzyme, in which the exonuclease domain encompasses amino acids 171–440, the linker region covers aa 476–785, and the polymerase domain is split into two and comprises amino acids 441–475 and 786–1239 (Lee et al. 2009). The exact domain sequence boundaries are still subject to dispute.

Mutations in the *POLG* gene may disrupt mtDNA replication, leading to the formation of multiple mtDNA deletions, to depletion (pathological decrease of the mtDNA copy number), or to a combination of these defects, which in turn disrupts the mitochondrial function and energy production (Stumpf and Copeland 2011). *POLG* mutations can, therefore, lead to a spectrum of mitochondrial diseases with dominant or recessive type of inheritance, which can be divided into three main groups: sporadic/familial progressive external ophthalmoplegia (PEO) (OMIM #157640 and #258450), adult-onset ataxia (OMIM #607459), and Alpers syndrome (OMIM #203700). Mitochondrial diseases attributed to mutations in *POLG* show a remarkable heterogeneity with, according to the Human DNA Polymerase Gamma Mutation Database (http://tools.niehs.nih.gov/polg/), more than 200 variants known to date, many of them rare.

The budding yeast is widely used as a genetic model system to study mtDNA stability and mutagenesis. Unlike most other microorganisms, *S. cerevisiae* can stably exist both in a haploid and a diploid state. Recessive mutations can be conveniently isolated and manifested in haploid strains, while complementation tests can be performed in the diploids. Heterozygous mutants allow the effects of dominant alleles to be observed. The most useful feature of these microorganisms in the study of mitochondrial function is that respiratory deficient mutants are viable and the presence of mtDNA is optional. The respiratory deficient phenotype can be easily observed as a formation of the so-called *petite* colonies on fermentative media, and lack of growth on respiratory-only media (Ephrussi and Slonimski 1955). *S. cerevisiae* is, therefore, an excellent model for the study of communication between nuclear and mitochondrial genetic systems. Due to the evolutionary conservation of some features of the mitochondrial system, like the mtDNA polymerase, they also may be used as a model for research on human mutations and their role in mtDNA replication (Barrientos 2003; Baruffini et al. 2007; Foury and Kucej 2002; Graziewicz et al. 2006; Longley et al. 2005; Stumpf and Copeland 2011; Lodi et al. 2015).

The yeast mitochondrial DNA polymerase (Mip1) is a nuclear-encoded protein, essential for the maintenance of the mitochondrial genome. It is orthologous to the human polymerase γ, and in spite of the evolutionary distance, its domain structure and amino acid sequence are essentially conserved (Foury 1989; Ropp and Copeland 1996). A yeast strain with its native mitochondrial DNA polymerase replaced by the human polymerase γ is viable and even maintains the functional mitochondrial genome essentially as efficiently as the wild type yeast (Qian et al. 2014). The Mip1 protein consists of four domains: the exonuclease-like domain, the linker region, the polymerase-like domain and the C-terminal domain. The polymerase and exonuclease domains are highly similar to their human equivalents. Unlike human Pol γ, Mip1 does not have any accessory subunits, and does not form a heterotrimer complex (Lucas et al. 2004; Yakubovskaya et al. 2006). Consequently, the linker region is not well conserved, and its sequence shows no significant similarity to that of the human ortholog. Another difference is the presence in Mip1 of a C-terminal domain, absent in human POLG (Viikov et al. 2011, 2012). In spite of those differences between yeast and human mtDNA polymerases, it is still possible to model many of the polymerase γ mutations found in patients with mitochondrial diseases in the yeast protein. As it is with POLG, even a single amino acid substitution in one of the conserved domains of Mip1 can lead to a severe mitochondrial deficiency phenotype. Combining comparable phenotypes with easy and fast analysis makes budding yeast an excellent model for examining pathologies of human polymerase γ (Barrientos 2003; Baruffini et al. 2006, 2007, 2011, 2014; Baruffini and Lodi 2010; Foury and Kucej 2002; Lodi et al. 2015; Stuart et al. 2006; Stricker et al. 2009; Stumpf et al. 2010; Stumpf and Copeland 2011, 2013, 2014; Szczepanowska and Foury 2010).

In search of new possibly pathogenic variants which can be modelled in *S. cerevisiae, POLG* sequence analysis was conducted for 60 patients with mitochondrial disease. Known well-characterised variants and novel sequence changes located in regions poorly conserved between human and yeast were rejected. We chose four previously undescribed variants for further study. The c.2604 C>T (p.Arg869Ter), c.2901C>G (p.Gln968Glu), c.3155_3156delCA (p.Thr1053Argfs*6) and c.3316 T>C (p.Val1106Ala) are located in the polymerase domain. In addition, one previously known substitution c.924C>T (p.Arg309Cys) located in the exonuclease domain was also included in the study. This mutation was reported once and only vaguely mentioned in the original work, additionally symptoms observed in both patients differ (Amiot et al. 2009). A novel nonsense mutation p.Arg869Ter in the polymerase domain serves as a control of the usefulness of our yeast model.

## Materials and methods

### Population analysis of novel POLG mutations

Analysis of the presence of the mtDNA deletions was conducted by long range PCR as described previously (Kierdaszuk et al. 2009). For patients with confirmed multiple mitochondrial DNA deletions, depletion or strong clinical indication, we performed *POLG* sequence analysis similar to (Filosto et al. 2003) (primer sequences are provided in Online Resource 1).

Reactions were performed in 25 µl final volume containing 0.16 µM of each of the forward and reverse primer, 0.4 mM of dNTPs, 1 U of Taq polymerase (A&A Biotechnology) and 100 ng of DNA. PCR conditions were 94° for 5 min, followed by 35 cycles of 94° for 1 min, appropriate annealing temperature (64° for POLG1_1, 6, 7, 8, 9, 10 C and 58° for POLG1_2, 3, 4, 5, 11, 12) for 1 min, 72° for 1 min and final elongation at 72° for 7 min. Sequencing was performed in the Laboratory of DNA Sequencing and Oligonucleotide Synthesis, Institute of Biochemistry and Biophysics, Polish Academy of Sciences. Sequences were compared with the *POLG* reference sequence (RefSeq NG_008218.1). The novel sequence variants were deposited in GenBank with accession numbers KP783508 (p.Gln968Glu), KP783509 (p.Arg290Cys), KP783511 (p.Arg869Ter), KP783513 (p.Val1106Ala), and KP783512 (p.Thr1053Argfs*6). We have also deposited sequence for p.Arg309Cys (KP783510) variant since it was not present in the GeneBank.

To estimate the frequency of the analysed *POLG* sequence variants in the Polish population, we designed and performed PCR–RFLP or high-resolution melting (HRM) tests on control DNA samples isolated from adults without neuromuscular disorders who gave their informed consent for genetic testing. HRM is a technique based on real-time PCR which utilizes differences in melting temperature of amplicons based on their nucleotide composition and enables to distinguish samples even with single-nucleotide substitution (Montgomery et al. 2010). Control samples for which test results were inconclusive were subsequently sequenced. In the case of p.Arg309Cys, a restriction site for the *Hha*I enzyme disappears in the POLG1_3 fragment, whereas for p.Thr1053Argfs*6 and p.Val1106Ala restriction sites for *Bae*GI and *Hinc*II, respectively, disappear in the POLG1_10 fragment. The digestion reactions were performed as follows: 5 µl of PCR product, 10 U of enzyme (New England Biolabs), 2 µl of buffer 2 (*Hha*I) or 3.1 (*Bae*GI and *Hinc*II), water up to 20 µl and incubated overnight in 37°.

To test the population frequencies of variants p.Arg869Ter and p.Gln968Glu, two pairs of primers for HRM test were designed (HRM869F/R and HRM968F/R, respectively). HRM reactions were prepared in 10 µl final volume containing MasterMix 1x (Roche), 3 mM (p.Arg869Ter) or 3.5 mM (p.Gln968Glu) MgCl_2_, 0.15 µM of each of the forward and reverse primer, and 60 ng of DNA. Initial denaturation for 10 min at 95° (4.8°/s) was followed by 45 cycles of 10 s at 95° (4.8°/s), 15 s at 60° (p.Arg869Ter) or 67° (p.Gln968Glu) (2.5°/s), 20 s at 72° (4.8°/s). Melting curves were determined as follows: 95° for 5 min (4.4°/s), 40° for 3 min (2.2°/s), 60° for 1 s (1°/s) and heating up to 95° (0.02°/s) with 25 acquisitions per 1°.

To verify reciprocal *in trans* localization of variants p.Arg309Cys and a second undescribed variant in patient AI2, we cloned a *POLG* fragment using primers H3POLG1_3F and S1POLG1_3R and pGEM-T Easy Vector into XL10-Gold chemocompetent *E. coli* (Stratagene).

### Yeast strains, plasmids and media

*S. cerevisiae* strains used in the research were two derivatives of W303, differing in the mating type: W303-1B (Chiron et al. 2005) (*MATaade2*-*1 leu2*-*3, 112 ura3*-*1 trp1*-*1 his3*-*11, 15 can1*-*100*) and CW04 (Chiron et al. 2005) (*MATαade2*-*1 leu2*-*3, 112 ura3*-*1 trp1*-*1 his3*-*11, 15 can1*-*100*). Strain Δmip1 was derived from CW04 by deleting the *MIP1* gene with the KanMX4 cassette (*MATa ade2*-*1 leu2*-*3, 112 ura3*-*1 trp1*-*1 his3*-*11, 15 can1*-*100 mip1::KanMX4*). D273-10B/51 (*MATαade5,* [*rho*^*0*^]) (Groudinsky et al. 1981) was used as a tester strain for the mitochondrial genome integrity.

The wild-type *MIP1* gene with approximately 300 bp of upstream and downstream sequences was amplified using primers mip1l and mip1r (Online Resource 1) and cloned into centromeric plasmids YCplac33 (*URA3)* and YCplac111 (*LEU2*) (Gietz and Sugino 1988) using the SLIC method (Li and Elledge 2007). The resulting constructs were named YCplac33MIP1 and YCplac111MIP1, respectively. Before transformation, all the constructs were verified by sequencing. For overexpression of mutant alleles, new constructs based on the pRS425 vector (Christianson et al. 1992) were created, by subcloning mutated sequences using the SLIC method (Li and Elledge 2007). All the constructs were verified by sequencing.

The following media were used in this work: YP medium consisted of 1 % yeast extract (Bioshop) 2 % Peptone (Bioshop) and 40 mg/l adenine (Sigma). YP medium was supplemented with 2 % glucose (YPD) or 2 % glycerol (YPG). SC medium consisted of 6.7 g/l YNB without amino acids (Bioshop) supplemented with an adequate amount of drop-out mix (Formedium™) according to the manufacturer’s instructions. The SC media were supplemented with 2 % glucose or 2 % glycerol. For evaluating the petite frequency, YPDG medium was used: 1 % yeast extract (Bioshop), 2 % Peptone (Bioshop), 40 mg/l adenine, 2 % glycerol and 0.1 % glucose. YPAEG-ery and YPAEG-oli media were used to determine frequencies of point mutations in mutant strains; they consisted of: 1 % yeast extract (Bioshop) 2 % Peptone (Bioshop) and 40 mg/l adenine, 2 % glycerol and 25 mM potassium phosphate buffer (pH 6.5) and 3 g/l erythromycin (Sigma) or 3 mg/l oligomycin (Sigma), respectively. For solid media, 2 % agar (Bioshop) was added.

### Construction of *mip1* mutant strains

The *mip1* mutant alleles were obtained by site-directed mutagenesis of the wild-type *MIP1* gene cloned into YCplac111 (YCplac111MIP1), using the double primer method of Zheng et al. (2004). The following mutated alleles were created (Table 2): Arg265Cys, Arg672Ter, Arg770Glu, Arg770Gln, Thr809Ter, Val863Ala (see Online Resource 1 for primer sequences). The W303/Δmip1 diploid, obtained by mating W303-1B and *Δmip1* strains, was transformed with both the wild-type YCplac33MIP1 and a mutated version of YCplac111mip1 plasmids. This provided a functional wild-type *MIP1* allele, allowing to maintain mtDNA (provided by the W303-1B parental strain) in the *Δmip1* background. This diploid was subsequently sporulated, and haploid spores bearing both plasmids and the genomic *Δmip1* gene deletion were selected using replica plating (-Ura, -Leu, G418 selection media). Subsequently, the YCplac33MIP1 *URA3* plasmid carrying the wild-type *MIP1* gene was removed by counterselection on 5-FOA plates (Sikorski and Boeke 1991), leaving the mutated variant of Mip1p as the only polymerase γ in the cells.

When the amino acid in the human *POLG* sequence was different from the corresponding residue in yeast Mip1p, in addition to the allele with the wild-type yeast residue replaced with the mutated human *POLG* amino acid (putative pathological allele), a second strain was created, with the wild-type human residue introduced into the yeast sequence, resulting in the humanized allele. The activity of the mutated protein was then compared both to the wild-type yeast sequence, and to the humanized variant of Mip1p.

### Determining the effect of mutations on oxidative growth phenotype

To define oxidative growth phenotype, 10 μl of 10^−1^, 10^−2^, 10^−3^ and 10^−4^ dilutions of liquid culture of each strain (starting from OD_600_ = 1) was spotted on YPD and YPG plates and incubated at 30° (normal temperature) or 37° (elevated temperature) for 3 days.

### Determination of mtDNA stability in yeast *mip1* mutants

Single colonies of strains obtained in the 5-FOA counterselection procedure were grown in liquid YPD medium at 30° or 37°. After 48 h of incubation, cells were diluted to OD_600_ = 1 and around 300–500 colonies were plated on YPDG medium. Colonies were incubated for 60 h at 30° and 37°. Subsequently, both *petite* and *rho*^+^ colonies were counted using the tetrazolium test (Ogur et al. 1957) (using 2 mg/ml of TTC).

For heteroallelic strains, appropriate selective SC medium with 2 % glucose instead of YPD was used for initial incubation. This was necessary to prevent the loss of plasmids in the preculture.

For each strain, at least 20 independent experiments were performed. Statistical significance of observed differences was evaluated using the *T* test (two-tailed, unequal variance) for *p* < 0.05 confidence levels.

To confirm that the respiratory deficient phenotype was due to changes in mtDNA, the *petite* strains were crossed to a wild-type *rho*^*0*^ tester strain (D273-10B/51), and the resulting diploids assayed for respiratory growth on YPG plates.

### Detection of mtDNA in yeast *petite* cells

The presence of mtDNA in yeast strains displaying the *petite* phenotype was assessed by staining of DNA in fixed cells with the Hoechst 3324 dye (Invitrogen), followed by fluorescent microscopy imaging as described previously (Puchta et al. 2010).

### RT-PCR for *MIP1* mRNA in yeast

Total cellular RNA was extracted according to the method described previously (Schmitt et al. 1990). RNA sample was treated with DNAse (Roche) in the presence of RNAse inhibitor (Ribolock, Thermo) in the buffer recommended by the manufacturer. DNA-free RNA was then extracted with phenol/chloroform/octanol (25:24:1), precipitated from the aqueous phase by ethanol/sodium acetate (pH 5.2) and dissolved in water. 5 μg of DNA-free RNA was reverse transcribed by Maxima™ Reverse Transcriptase (Thermo Scientific) according to the manufacturer’s instructions. Reactions without reverse transcriptase (RT-) were used as controls to test for genomic DNA contamination. cDNA was then amplified by PCR using the rtMipL and rtMipshR or rtMipL and rtMiploR primer pairs (Online Resource 1).

### Quantification of point mutation accumulation in yeast mtDNA

To evaluate point mutation frequencies, two independent series of experiments consisting of 10 repeats were conducted. Each colony was grown in 3 ml of YPG for 48 h at 30°. Subsequently, 6–8 × 10^7^ colonies were plated on YPAEG-ery medium and incubated at 30° for 9 days. Simultaneously, for each YPG culture, 50 μl of a 10^−5^ dilution was plated on a YPG plate to establish the exact number of colony-forming units in the culture.

The mutation frequencies in Table 2 are shown as the mean count of Ery^R^ colonies per 10^8^ CFU. The Mann–Whitney *U* test was used to establish statistical significance for this experiment.

For the Oli^R^ experiment, the same protocol was used, except that instead of 3 g/l erythromycin, 3 mg/l oligomycin was added to the medium and the number of colonies plated on YPAEG-oli plate was approximately 3–5 × 10^7^ CFU.

## Results

### Novel POLG variants in mitochondrial disease patients

In a search for previously undescribed variants, *POLG* coding sequence analysis was conducted for 60 patients with clinical diagnosis of myopathy (OMIM # 251900) (*n* = 18), encephalomyopathy (*n* = 5), PEO (OMIM #157640 and #258450) (*n* = 4), PEO and myopathy (*n* = 3), encephalopathy (*n* = 3), polyneuropathy (*n* = 2), myoclonic epilepsy associated with ragged-red fibres (MERRF) (OMIM #545000) (*n* = 2), Kearns-Sayre syndrome (OMIM #530000) (*n* = 1), myopathy, encephalopathy, lactic acidosis and stroke-like episodes (MELAS) (OMIM #540000) (*n* = 1), mitochondrial neurogastrointestinal encephalopathy (MNGIE) (OMIM #613662) (*n* = 1), sensory ataxic neuropathy, dysarthria and ophthalmoparesis (SANDO) (OMIM #607459) (*n* = 2), general suspicion of mitochondrial disease (*n* = 15) or Alpers syndrome (OMIM #203700) (*n* = 3). Sequencing of the *POLG* gene in these patients, followed by in silico translation, revealed 1 poorly uncharacterised and 5 previously unreported sequence changes located in the regions conserved between yeast and human (c.924 C>T/p.Arg309Cys and c.867 C>T/p.Arg290Cys—proband from family A, c.2604 C>T/p.Arg869Ter—proband from family B, c.2901 C>G/p.Gln968Glu—proband from family C, c.3155_3166delCA/p.Thr1053Argfs*6—proband from family D and c.3316 T>C/p.Val1106Ala—proband from family E). None of the novel variants were present in the Human Polymerase Gamma Mutation Database, NCBI dbSNP (Sherry 2001), the 1000 genomes project (Genomes Project Consortium 2012), or in the Exome Variant Server database (http://evs.gs.washington.edu/EVS/). These variants, along with the poorly characterised p.Arg309Cys, were therefore selected for further investigation, and their sequences deposited in GenBank (accession numbers are listed in “Materials and methods”).

### Patients’ clinical presentation

**Fig. 1:**
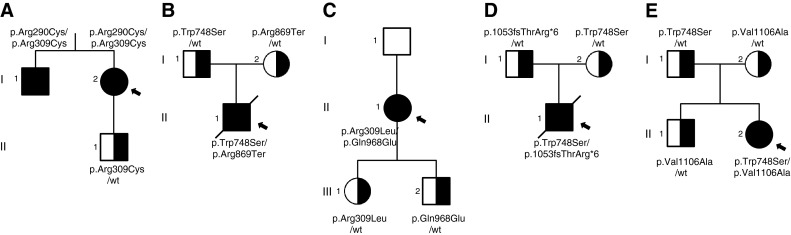
*POLG* variant distribution analysis in patients and their families. **a** Proband (I2) and her brother (I1) are both affected and have one known, but poorly characterised and one novel variant (p.Arg290Cys/p.Arg309Cys), whereas the proband’s healthy son (II1) is a carrier of the p.Arg309Cys variant. **b** Proband (II1) died at the age of 2.5 years due to liver failure caused by valproic acid administration. He carried heterozygous p.Trp748Ser mutation and heterozygous novel variant p.Arg869Ter. His parents (I1 and I2) are heterozygous carriers of either pathogenic mutation p.Trp748Ser (I1), or the novel variant p.Arg869Ter (I2). **c** Proband (II1) carries heterozygous pathogenic mutation p.Arg309Leu and heterozygous novel variant p.Gln968Glu. Her children (III1 and III2) are unaffected heterozygous carriers of pathogenic mutation p.Arg309Leu (III1), and the novel variant p.Gln968Glu (III2). The presence of p.Gln968Glu in the proband’s father (I1) was excluded, but due to the lack of biological material we were unable to perform the analysis for the presence of the p.Arg309Leu mutation. **d** The proband (II1) died at the age of 3.5 years after liver failure due to valproic acid administration. Both parents (I1 and I2) have either the novel p.Thr1053Argfs*6 (I1) variant or the known pathogenic mutation p.Trp748Ser (I2). **e** Proband (II2) carries heterozygous pathogenic mutation p.Trp748Ser together with heterozygous novel variant p.Val1106Ala. Her father (I1) is a carrier of the known pathogenic mutation p.Trp748Ser, whereas both her mother (I2) and brother (II1) have heterozygous novel variant p.Val1106Ala. All heterozygous family members are asymptomatic. Since the analysis of familial distribution of *POLG* variants present in families **a**–**e** indicates that all the probands are compound heterozygotes *in trans*, an autosomal recessive mode of inheritance is suggested

**Fig. 2:**
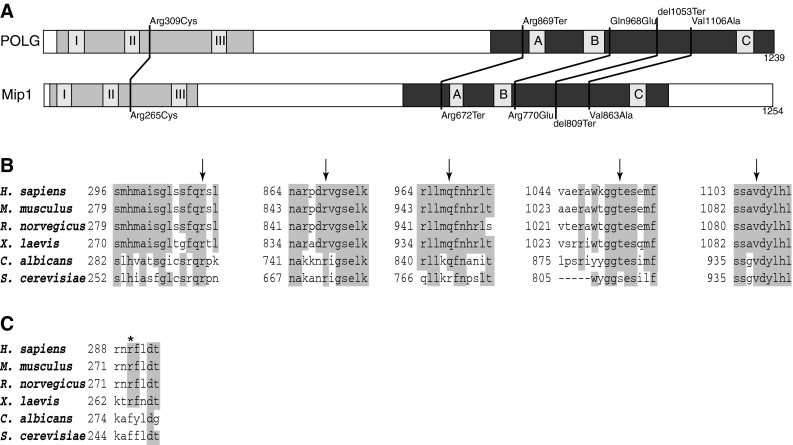
Modelling human POLG variants using the *S. cerevisiae* ortholog—Mip1p. **a** Schematic representation of POLG and Mip1 protein primary structure, with locations of the studied human POLG variants and the corresponding residues in *S. cerevisiae* Mip1p. The exonuclease domain is shown *shaded light grey* and the polymerase domain is *shaded dark grey*. Sequence of the linker region is not conserved. Blocks *I*, *II*, and *III* in the exonuclease domain, and *A*, *B*, and *C* in the polymerase domain are the regions of highest sequence conservation. **b** Alignment of the mitochondrial DNA polymerase amino acid sequences of selected vertebrate and yeast species in the regions of human variants selected for yeast modelling. Conserved residues (>0.5 consensus identity) are* highlighted,* *arrows point* to residues changed in the variant alleles under study. **c** Alignment of the mitochondrial DNA polymerase amino acid sequences of selected vertebrate and yeast species in the region around human Arg290 residue (marked with an *asterisk*), where lack of conservation between vertebrate and yeast sequences makes modelling of the Arg290Cys variant impractical

The results of sequence analysis, clinical and molecular evaluation of the four patients carrying previously unreported *POLG* variants and one with the known, but poorly characterised variant, are summarized in Table 1. Proband from family A (AI2) (Fig. 1a) is a 54-year-old woman with SANDO syndrome and positive family history (her brother presented similar symptoms). From the age of 31, she suffered from progressive external ophthalmoplegia, ptosis, dysarthria, weakness of upper and lower limbs and sensory ataxic neuropathy. Additionally, mental retardation was diagnosed. Nerve conduction studies indicated axonal sensory and motor neuropathy. MRI showed brain atrophy. Skeletal muscle biopsy disclosed ragged-red fibres on light microscopy examination. Analysis of mitochondrial DNA revealed multiple deletions in muscle tissue. Along with the p.Arg309Cys variant, patient AI2 also had another previously unknown sequence change c.867 C>T (p.Arg290Cys) for which, due to a low level of similarity in this position between POLG and Mip1 (Fig. 2c), creating a yeast model was impractical. According to PolyPhen-2 (Adzhubei et al. 2010), a software tool which predicts possible impact of a single amino acid substitution on the function of human proteins, both these variants found in patient AI2 are probably damaging, with a score of 1.00. SIFT Human Protein DB tool also predicts that both variants p.Arg290Cys and p.Arg309Cys are not tolerated (SeqRep = 0.92) (Ng and Henikoff 2001). The proband’s brother (AI1) carries both p.Arg309Cys and p.Arg290Cys variants present in the proband AI2.

**Table 1:** Clinical symptoms, mtDNA and *POLG* sequence analysis in patients

Proband	*POLG* sequence variants^a^	Result of mtDNA analysis	Clinical diagnosis
AI2	**c.867 C>T (p.Arg290Cys)**/c.924 C>T (p.Arg309Cys)	Multiple deletions^b^	SANDO
BII1	c.2243 G>C (p.Trp748Ser)/**c.2604 C>T (p.Arg869Ter)**	Depletion^c^	Alpers syndrome
CII1	c.925 G>T (p.Arg309Leu)/**c.2901 C>G (p.Gln968Glu)**	Multiple deletions^b^	PEO
DII1	c.2243 G>C (p.Trp748Ser)/c.3155_3156delCA (p.Thr1053Argfs*6)	Depletion^c^	Alpers syndrome
EII2	c.2243 G>C (p.Trp748Ser)/**c.3316 T>C (p.Val1106Ala)**	No lesions detected	SANDO

Bold text indicates novel variants

^a^Compared to the *POLG* reference sequence (RefSeq NG_008218.1)

^b^Muscle tissue

^c^Liver tissue

The proband from family B (BII1) was without any symptoms at infancy. At the age of 12 months, he became progressively ataxic and recurrent convulsions appeared, developing quickly to severe epileptic encephalopathy. Soon after introduction of anticonvulsant valproic acid administration, a fulminant liver failure was observed and the patient died at the age of 2.5 years. Severe mitochondrial depletion found in the liver tissue on autopsy (0.08 %, while the threshold for diagnosing depletion is set at <30 % of the normal mtDNA level) was in agreement with the clinical suspicion of Alpers syndrome. *POLG* sequencing revealed two *in trans* variants, one known pathogenic p.Trp748Ser substitution (Palin et al. 2010; Van Goethem et al. 2004), and a novel variant p.Arg869Ter.

Proband from family C (CII1) (Fig. 1c) is a 53-year-old woman with progressive external ophthalmoplegia and negative family history. At the age of 38, she observed impaired eye movements and ptosis. Nerve conduction studies were normal, and electromyography indicated myogenic changes. Cardiological assessment revealed no abnormalities. Skeletal muscle biopsy disclosed ragged-red fibres on light microscopy, and multiple abnormal mitochondria with paracrystalline inclusions on electron microscopy examination. Multiple mtDNA deletions were present in muscle tissue. *POLG* sequencing revealed a known p.Arg309Leu (Lamantea et al. 2002) mutation and a novel variant p.Gln968Glu, predicted to be not tolerated by SIFT (SeqRep = 0.97), and probably damaging with a score of 0.992 according to PolyPhen2.

In the proband from family D (DII1) (Fig. 1d), a psychomotor retardation and muscle hypotonia were observed since 2 months of age, and autistic behaviour was observed during early chilhood. At the age of 3 years, the patient was given valproic acid due to episodes of unconsciousness and abnormal EEG pattern and responded suddenly with an acute liver failure. He died 6 months later before the qualification for liver transplantation was completed. Severe depletion of mtDNA was found in the liver section taken at autopsy (3.4 %, ref. >30 %), as in the case of the patient BII1. In the *POLG* sequence, in addition to the known p.Trp748Ser mutation, a novel variant p.Thr1053Argfs*6 was found.

In spite of normal developmental milestones, the proband from family E (EII2) (Fig. 1e) started to have walking difficulties at the age of 13 years. Her condition deteriorated, and she developed ataxic gait and dysarthria. Two years later, she developed action-exacerbated myoclonus. When last seen at 15 years, she had no ophthalmoparesis. Nerve conduction studies showed sensory-motor polyneuropathy of lower limb nerves. We did not detect mtDNA deletions in a DNA sample isolated from peripheral blood. Since muscle tissue was not available, the decision to sequence *POLG* was undertaken based on the patient’s clinical presentation, and indicated that she was a compound heterozygote p.Trp748Ser/p.Val1106Ala. The novel variant (p.Val1106Ala) is designated as not tolerated (SIFT SeqRep = 0.98) and possibly damaging with a score of 0.85 (PolyPhen2). Interestingly, another amino acid change (valine to isoleucine) in the same position was described previously as probably pathogenic (Horvath et al. 2006).

Each of the novel variants coexisted in the patient with a known pathogenic recessive mutation, and all the patients’ parents were healthy; therefore, we suspected that all the variants, if pathogenic, were also recessive. For all the patients, DNA samples isolated from other members of their families were also available. To confirm *in trans* localization of novel versus known variants in patients AI2, BII1, CII1, DII1 and EII2, we performed family studies, and in one case cloning.

### Familial and population study

To verify reciprocal localization of novel and known variants in patients, we acquired DNA samples from their family members, and amplified and subsequently sequenced the relevant *POLG* fragments (Fig. 1). In three families (B, D and E), the proband’s parents were each carrier of one of the two pathogenic/possibly pathogenic variants. In the case of proband AI2, the parents’ DNA was unavailable, but *POLG* sequence analysis of her son (AII1) proved him to be the carrier of the known, but poorly characterised p.Arg309Cys variant. Her symptomatic brother (AI1) had both variants p.Arg290Cys and p.Arg309Cys found in the proband. Additionally, we performed cloning of *POLG* alleles in patient AI2, which further confirmed that she was a compound heterozygote. The family of patient CII1 did not give consent to obtain their DNA from peripheral blood, but agreed to perform buccal swabs. Both her children are heterozygous carriers of the pathogenic (CIII1-p.Arg309Leu) or possibly pathogenic (CIII2-p.Gln968Glu) variant. The presence of the novel variant p.Gln968Glu in her father (CI1) was ruled out, but due to a low yield of DNA extraction, we were not able to confirm the presence of the p.Arg309Leu mutation. Thus, we confirmed that in all the affected individuals, the two variants were *in trans*.

The population frequency of all novel variants was estimated using PCR–RFLP or HRM tests. Because the population frequency of p.Arg309Cys was not previously assessed, we also conducted PCR–RFLP test for this variant. None of the analysed *POLG* sequence variants were found in 320 chromosomes of healthy adults, which means that the population frequency of each variant is significantly lower than 1 %.

The analysis of familial distribution and frequency in the general population suggests that the analysed *POLG* sequence changes are probably pathogenic and inherited in a recessive manner.

### Modelling the putative pathogenic mutations in yeast

To gain some insights into the effects of the novel variants in the human polymerase γ using the well established and accessible *S. cerevisiae* model, we first aligned the amino acid sequences of human *POLG* and its yeast ortholog Mip1p, and identified the residues that were mutated in patient sequences (Fig. 2). Of the 5 residues, three (Arg309, Arg869, and Val1106) were conserved in the yeast Mip1p. The site of the frameshift mutation p.Thr1053Argfs*6 also corresponds to a block of a nearly complete amino acid sequence identity. The Gln968 residue, also in a conserved region, corresponds to Arg770 in the yeast protein. In this case, in addition to the mutated allele (with Glu in this position), we also created the humanized allele (Arg770Gln).

The mutant *mip1* alleles were created by site-directed mutagenesis and introduced into the *Δmip1* background on centromeric (low copy number) vectors, as described in “Materials and methods”. The plasmid shuffling strategy was used to maintain functional mtDNA owing to the presence of the wild-type *MIP1* gene on an *URA3* vector, which could subsequently be eliminated by 5-FOA counterselection (Sikorski and Boeke 1991).

### Influence of *mip1* mutations on respiratory growth phenotype in the yeast model

**Fig. 3:**
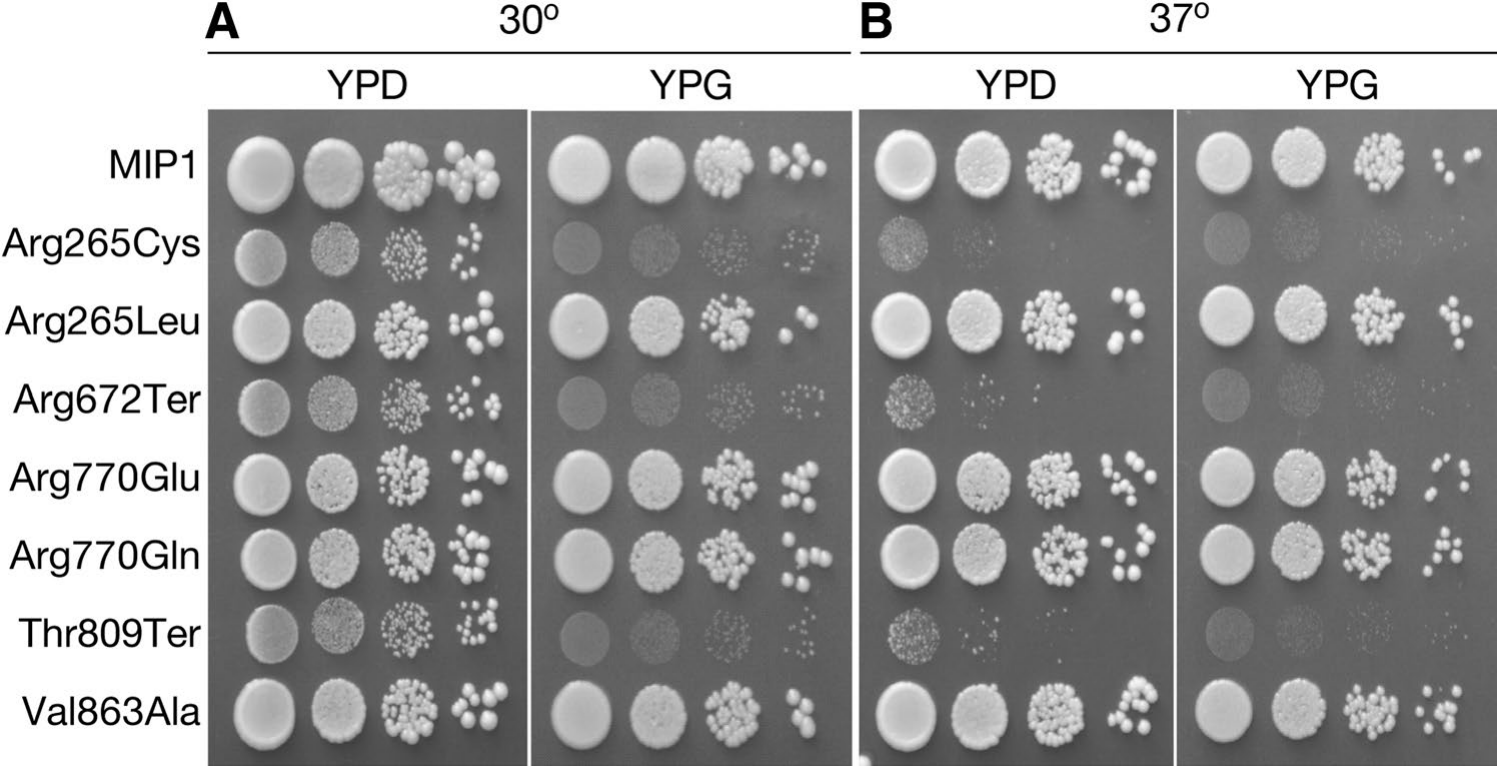
Respiratory growth of *S. cerevisiae* strains bearing the wild type and mutated alleles of *MIP1*. Fermentative growth was tested on YPD medium (YP with 2 % glucose) at 30° and 37°. Respiratory growth was tested by plating the strains on YPG medium (YP with 2 % glycerol) and incubation at 30° and 37°. Each strain was spotted in 10^−1^, 10^−2^, 10^−3^ and 10^−4^ dilutions (starting from OD_600_ = 1). The pictures were taken after 3 days of incubation. **a** Fermentative (YPD) and respiratory (YPG) growth at normal temperature (30°). All the strains grow on the fermentable carbon source (YPD), although the strains bearing the Arg265Cys, Arg672Ter and Thr809Ter variants show slightly slower growth. These strains are not able to grow on medium with the nonfermentable carbon source (YPG) (**b**). The same growth assay as in (**a**), but carried out at the elevated temperature (heat stress, 37°). The phenotype of strains bearing the Arg265Cys, Arg672Ter and Thr809Ter variants is even more pronounced at the elevated temperature, and the growth defect on the fermentable carbon source is more apparent. The strains that are not affected at normal temperature do not show a respiratory deficiency at 37° either

Growth on a non-fermentable carbon source (glycerol or ethanol) is the fastest and simplest method to examine respiratory function in *S. cerevisiae* strains. Among the five tested strains carrying yeast equivalents of putative pathogenic mutations, three exhibited a severe respiratory deficiency. Mutants Arg265Cys, Arg672Ter and Thr809Ter were unable to support growth on glycerol at 30° (Fig. 3). Growth of the remaining strains did not differ from the wild-type. Incubation at a restrictive temperature (37°) contributed to an even more severe phenotype of mutants which were unable to grow on glycerol (Arg265Cys, Arg672Ter, Thr809Ter), causing slower growth even on a medium with fermentable carbon source. The elevated temperature did not significantly affect the rest of the tested strains.

### Determination of *petite* frequencies in strains with *mip1* mutations

*Petite* mutants of *S. cerevisiae* are unable to grow on non-fermentable carbon sources and form only small anaerobic colonies in the presence of glucose. While this phenotype can result from a point mutation in mtDNA or a mutation in a nuclear gene, most often it is caused by a complete loss (*rho*^0^) or extensive deletions (*rho*^−^) of mtDNA. *Petite* colonies appear at a low (1–5 %) rate spontaneously in cultures grown on fermentable carbon sources. Mutations in nuclear genes encoding proteins involved in mitochondrial function often result in a marked increase in the frequency of *petite* generation (Contamine and Picard 2000; Lipinski et al. 2010).

**Fig. 4:**
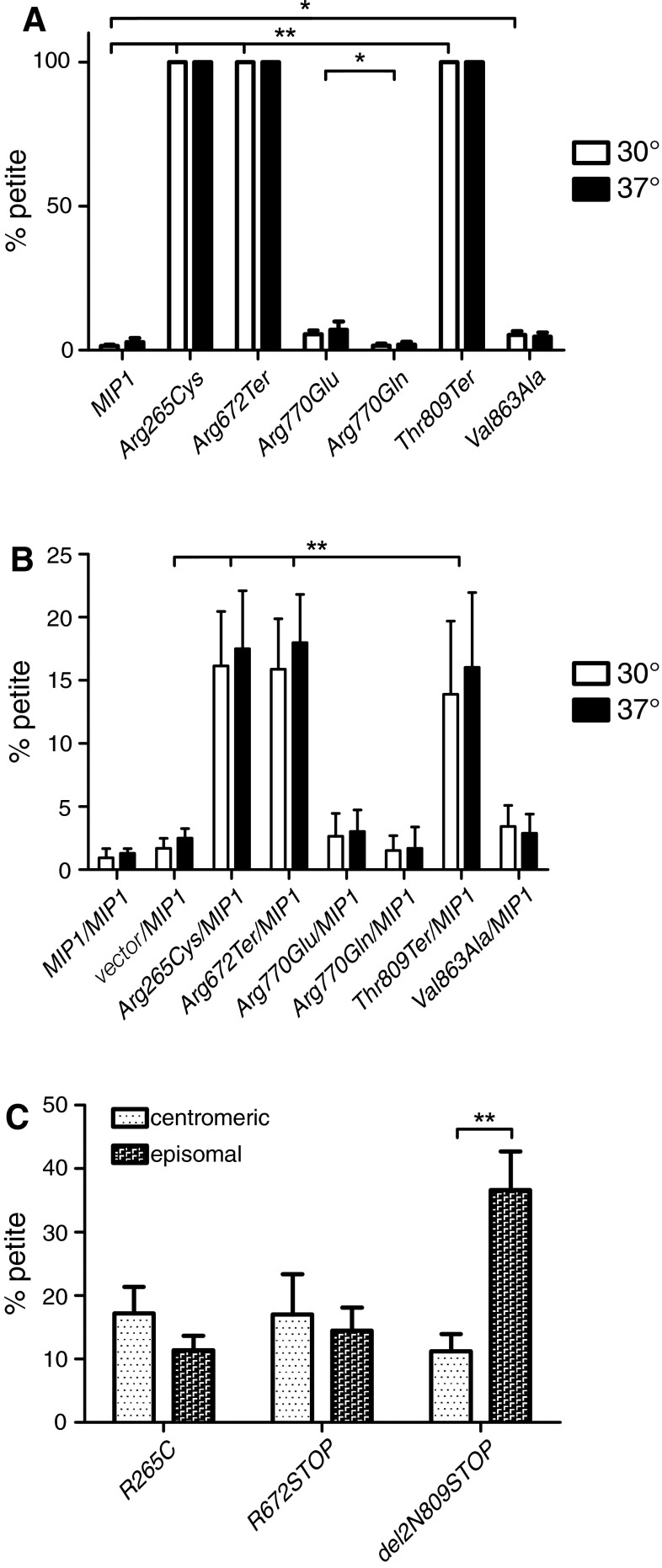
Mitochondrial genome stability in yeast strains carrying *MIP1* alleles corresponding to the *POLG* variants found in human patients. **a** Frequencies of *petite* mutants in strains carrying the wild-type *MIP1* or mutated *mip1* alleles in the homoallelic setting (as the only *MIP1* allele on a centromeric plasmid). Mutations Arg265Cys, Arg672Ter, and Thr809Ter lead to a complete loss of functional mtDNA (100 % *petites*) and the *petite* frequency in Mip1Val863Ala strain is three times higher than in the wild type strain. The Arg770Glu mutation significantly increases *petite* accumulation in comparison to either the wild type, or humanized (Arg770Gln) allele. There is no significant difference between the humanized Arg770Gln and the wild-type allele. **b** Frequencies of *petite* mutants in the pure wild-type (*MIP1/MIP1*), hemiallelic (vector/*MIP1*) and heteroallelic (*MIP1/mip1*) strains, with both alleles expressed from low copy number (centromeric) plasmids. In heteroallelic strains with Arg265Cys/*MIP1*, Arg672Ter/*MIP1* and Thr809Ter/*MIP1* alleles, *petites* accumulate at a significantly higher frequency than in the hemiallelic (vector/*MIP1*) strain; nevertheless, they still retain >80 % of functional mtDNA. *Petite* frequency in the Val863Ala/*MIP1* shows a slight (but significant at *p* < 0.05) difference compared with the hemiallelic (vector/*MIP1*) at 30°, but not at 37° (not marked on the graph). The Arg770Glu/*MIP1* heteroallelic strain shows no significant difference compared to either the hemiallelic (vector/*MIP1*) or the humanized strain. Only the strongly significant differences (*p* < 0.01) were marked with *asterisks* in this panel for clarity. **c** Frequencies of *petite* mutants resulting from the overexpression (on the multicopy pRS425 plasmid) of mutant alleles (in the background of the wild-type *MIP1* on a low copy number vector). For Arg265Cys and Arg672Ter variants, no significant difference was observed between the low copy number and multicopy vectors, but for the Thr809Ter allele the *petite* frequency was significantly higher when the mutated allele was on a multicopy plasmid, suggesting an antimorphic character of the mutation. The *T* test (two-tailed, unequal variance) was used to assess the statistical significance, *p < 0.05, and **p < 0.01. *ns* not significant. Numerical data can be found in Table 2

All the strains carrying *mip1* alleles corresponding to the modelled human mutations show increased *petite* frequencies in comparison to the one with the wild-type *MIP1* (Fig. 4a; Table 2). Furthermore, mutations Arg265Cys, Arg672Ter, Thr809Ter, which cause a complete respiratory deficiency, lead to a complete loss of functional mtDNA in the tested strains.

**Table 2:** Analysis of mtDNA stability and point mutations in mutant yeast strains carrying *MIP1* alleles corresponding to the *POLG* variants found in human patients

Variant in *H. sapiens*	Mutation in *S. cerevisiae*	Domain	*Petite* frequency (%)				Oli^R^/10^8^CFU	Ery^R^/10^8^CFU
			Homoallelic		Heteroallelic with wt *MIP1*			
			30°	37°	30°	37°		
–	wt *MIP1*	–	1.4 ± 0.8	2.8 ± 1.5	1.0 ± 0.7	1.3 ± 0.4	0.7 ± 0.3	0.5 ± 0.18
	Hemiallelic vector/*MIP1*				1.7 ± 0.8	2.5 ± 0.7		
p.Arg309Cys	Arg265Cys	exo	100 ± 0**	100 ± 0**	16.2 ± 4.3**	17.5 ± 4.6**	–	–
p.Arg869Ter	Arg672Ter	pol	100 ± 0**	100 ± 0**	15.9 ± 4.0**	18 ± 3.8**	–	–
p.Gln968Glu	Arg770Glu	pol	5.6 ± 1.8*	7 ± 2.9*	2.7 ± 1.8	3.0 ± 1.7	15 ± 7.8*	7.4 ± 1.4*
	Arg770Gln (humanized)		1.6 ± 1.0	2.0 ± 0.9	1.5 ± 1.2	1.7 ± 0.5	3.8 ± 1.8	1.6 ± 0.33
p.Thr1053Argfs*6	Thr809Ter	pol	100 ± 0**	100 ± 0**	13.9 ± 5.8**	16.0 ± 5.9**	–	–
p.Val1106Ala	Val863Ala	pol	5.3 ± 1.7*	4.7 ± 1.4*	3.4 ± 1.7*	2.9 ± 1.5	3.6 ± 2.7*	3.4 ± 0.1*

Statistical significance calculated compared to the wild-type Mip1 for homoallelic strains, except for Arg770Glu, where the humanized Arg770Gln variant was used as reference

For the heteroallelic strains, statistical significance was calculated compared to the hemiallelic vector/*MIP1* strain

* *p* < 0.05, ** *p* < 0.01

For the Arg770Glu mutation, *petite* accumulation is significantly (*p* < 0.05) higher than in its humanized allele mip1Arg770Gln and in the wild-type version of the protein (5.6, 1.6, and 1.4 % at 30°, respectively). Furthermore, there is no significant difference between wild-type Mip1p (1.4 % petite) and humanized Arg770Gln (1.6 %). *Petite* frequency of the strain carrying the Val863Ala allele (5.3 %) is approximately three times higher than in the wild-type strain at 30°, and this difference is statistically significant (*p* < 0.05).

To confirm that the observed respiratory negative phenotype was related to the loss of functional mtDNA, *petite* colonies from the strains carrying the Arg265Cys, Arg672Ter and Thr809Ter variants (50 colonies each) were crossed to a wild-type *rho*^*0*^ tester, and the resulting diploids assayed for respiratory growth. This test confirmed that in all the *petite* colonies there was no functional mtDNA left (*rho*^−^*/rho*^*0*^). Due to the presence of nonfunctional residual mitochondrial genome fragments in *rho*^−^ mtDNA, it is not practical to perform quantitative analysis of mtDNA levels in yeast cells using PCR-based methods. For the strains carrying the Arg265Cys, Arg672Ter and Thr809Ter variants, DNA staining with Hoechst dye followed by fluorescent microscopy imaging revealed that, unlike in the wild-type control, extranuclear DNA signal is mostly absent, suggesting that they are essentially *rho*^*0*^ (Online Resource 2).

The severe phenotype of the Arg265Cys, Arg672Ter and Thr809Ter variants raised the possibility that these mutations abolished the expression of the *MIP1* gene, and thus were equivalent to transcriptional nullomorphs. We performed an RT-PCR assay on total RNA isolated from the strains carrying the Arg265Cys, Arg672Ter and Thr809Ter variants as the only *MIP1* alleles, and found no apparent change compared to the wild-type control, thus confirming that these mutations do not interfere with the expression of the *MIP1* gene, at least on mRNA level (Online Resource 3).

### Determination of *petite* frequencies in heteroallelic strains

To determine the dominance/recessivity of the mutations, we assayed the frequency of *petite* formation in heteroallelic yeast strains bearing each of the mutant alleles as well as the wild-type *MIP1* allele (Fig. 4b; Table 2). A hemiallelic strain containing the empty YCplac111 vector as well as the vector carrying the wild type *MIP1* (vector*/MIP1*) was also used as a control to normalise the allele dosage and differentiate between antimorphy and haploinsufficiency. The *petite* frequency in the hemiallelic strain is slightly, but significantly (*p* < 0.05) higher than in the *MIP1*/*MIP1* strain, and this effect is more pronounced at the elevated temperature (37°). *MIP1* haploinsufficiency is evident when a diploid strain heterozygous for the *Δmip1* allele is used (Baruffini et al. 2006). The haploid deletant strain with plasmid-borne wild-type and mutant *MIP1* alleles used in this study is clearly less sensitive to haploinsufficiency.

In the Val863Ala/*MIP1* strain, the *petite* frequency was slightly, yet significantly (*p* < 0.05) higher than in the vector*/MIP1* strain at 30°. No increase in *petite* frequency could be observed at the elevated temperature (37°). Even though the difference at 30° was statistically significant, the *petite* frequency in this heteroallelic strain remained low (well below 5 %); the dominant negative effect of this allele is thus negligible.

The *petite* accumulation frequency for the heteroallelic strain Arg770Glu/*MIP1* strain, as well as for the humanized Arg770Gln/*MIP1,* was not significantly elevated above the level observed in the hemiallelic vector*/MIP1* control at either temperature; this mutation is, therefore, recessive.

Strains heteroallelic for the three mutations that showed the most severe phenotype (complete respiratory deficiency and loss of functional mtDNA) in the monoallelic setting, Arg265Cys/*MIP1*, Arg672Ter/*MIP1*, and Thr809Ter/*MIP1*, accumulated *petites* at a much higher frequency than the hemiallelic vector*/MIP1* control strain (at both temperatures), although they still retained >80 % of functional mtDNA. These alleles thus have a partially dominant negative effect on the mtDNA replication process (Fig. 4b; Table 2). To verify whether this effect is dependent on allele dosage, we also constructed strains carrying the mutant alleles on multicopy (episomal) plasmids, and the wild-type allele on a centromeric plasmid (Fig. 4c). For Arg265Cys and Arg672Ter, no significant difference was observed between the low copy number and multicopy vectors, for the Thr809Ter allele; however, increasing the copy number of the mutated variant resulted in a significantly (*p* < 0.01) more pronounced phenotype of the heteroallelic strain, suggesting a true antimorphic character of this mutation.

### Frequency of mtDNA point mutations in the yeast model

Two of the five mutated *mip1* alleles, Arg770Glu and Val863Ala, showed only a partial decrease in overall mtDNA stability (measured by the frequency of *petite* colony formation), whereas the humanized Arg770Gln variant was indistinguishable from the wild type in that aspect. No effect on the respiratory growth was observed in these three strains. To test whether the replicative function of the mitochondrial DNA polymerase in these strains was not affected in a more subtle way, we performed experiments assessing the point mutation rates in mtDNA.

Quantification of point mutation accumulation in mtDNA is based on estimating the frequencies of spontaneous oligomycin- (Oli^R^) and erythromycin-resistant (Ery^R^) mutants that result from substitutions in mitochondrial genes encoding the ATPase subunits or the large subunit rRNA, respectively. This assay can indicate changes in the fidelity of mtDNA replication, such as defects in the proofreading activity of the mitochondrial DNA polymerase.

**Fig. 5:**
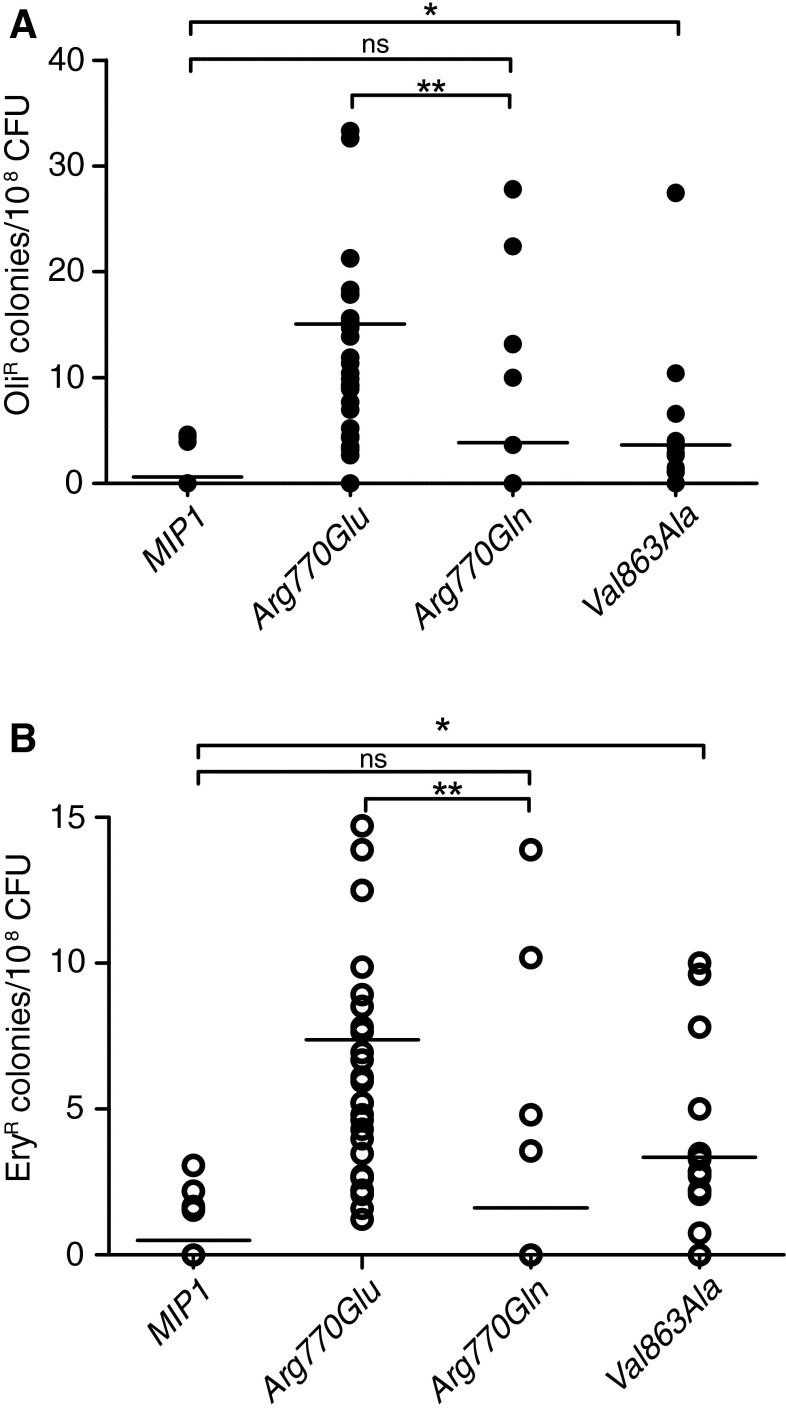
Replication fidelity, expressed as point mutation frequencies in yeast strains carrying *MIP1* alleles corresponding to the *POLG* variants found in human patients. Oligomycin- and erythromycin-resistant mutant frequencies were calculated as a fraction of the *rho* ^+^ cells plated on the respiratory (YPG) medium for the *mip1* alleles that did not cause a complete loss of mtDNA stability. **a** Frequencies of mutations (in mitochondrial genes encoding the ATPase subunits) resulting in oligomycin resistance. *Black circles* correspond to the antibiotic resistant colonies on individual plates. **b** Frequencies of mutations (in the mitochondrial gene encoding the large subunit rRNA) resulting in erythromycin resistance. *Open circles* correspond to the antibiotic resistant colonies on individual plates. *Horizontal lines* indicate the mean value of medians from two independent experimental repetitions (10 plates each). In both assays, the strain carrying the Val863Ala mutation showed a significant increase in the point mutation frequency. The effect of the Arg770Glu allele on mutation frequencies was even more pronounced, and significantly elevated either in comparison to the wild-type *MIP1,* or to the Arg770Gln humanized variant, whereas differences between the humanized variant and the wild-type allele were not statistically significant. Mann–Whitney *U* test was used to assess the statistical significance, *p < 0.05, and **p < 0.01. *ns* not significant. Numerical data can be found in Table 2

Two mutations (Arg770Glu, Val863Ala) and the humanized allele (Arg770Gln) were examined in comparison to the wild-type strain (Table 2; Fig. 5). The strain carrying the Val863Ala allele showed a slight, but still statistically significant (*p* < 0.05, Mann–Whitney *U* test) increase in the frequency of both Oli^R^ and Ery^R^ mutations. The effect of the Arg770Glu allele, on the other hand, was more pronounced, and significantly (*p* < 0.05) higher both in comparison with the wild-type *MIP1,* and with the Arg770Gln humanized variant (differences between the humanized allele and the wild type version were not significant). Interestingly, this substitution (as well as Val863Ala) is located in the polymerase domain (Table 2). It has to be noted, however, that the effect observed for this variant, while statistically significant (*p* < 0.05), is far less evident than that reported for an *MIP1* allele devoid of the corrective exonuclease domain activity (*exo*^−^), where the Ery^R^ mutation frequency increased by 550-fold (Stumpf et al. 2010).

## Discussion

To date, over 200 *POLG* sequence changes are known, and for the vast majority their pathogenicity remains unclear. Since most variants are classified as pathogenic only on the basis of their presence in the patient, there are only a few well-documented *POLG* mutations. Here, we present an in-depth analysis of 5 *POLG* variants—4 novel and 1 known but poorly characterised, found in patients with mitochondrial disorder symptoms: p.Arg309Cys, p.Arg869Ter, p.Gln968Glu, p.Thr1053Argfs*6 and p.Val1106Ala, combining familial and population studies with a functional study in a yeast model. Since the population frequency of all the analysed variants is lower than 1 %, it is highly probable that they are not polymorphic sequence changes but true pathogenic mutations.

As it is often the case for the severe recessive mitochondrial disorders caused by *POLG* mutations (like the Alpers syndrome) (Chan et al. 2009; Euro et al. 2011; Mancuso et al. 2004; Wiltshire et al. 2008; Wong et al. 2008), the affected individuals are compound heterozygotes. In our study, all the novel *POLG* variants in the patients coexist with another known mutation. Pedigree analysis, together with the results of the general population study, strongly suggests that p.Arg309Cys, p.Arg869Ter, p.Gln968Glu, p.Thr1053Argfs*6 and p.Val1106Ala are indeed pathogenic variants inherited in a recessive manner. To verify these assumptions, we used the yeast *S. cerevisiae* as a model for studying phenotypes of the 4 novel and 1 known but poorly characterised *POLG* mutations that occur in sequence regions conserved between yeast and human orthologs. The validity of using *S.* *cerevisiae* as a model in studying known human pathogenic *POLG* mutations was demonstrated in many cases (Barrientos 2003; Baruffini et al. 2006, 2007, 2011, 2014; Baruffini and Lodi 2010; Foury and Kucej 2002; Lodi et al. 2015; Stuart et al. 2006; Stumpf et al. 2010; Stumpf and Copeland 2011, 2013, 2014), including cases where no other evidence for causation was available (Stumpf et al. 2010), yet studies simultaneously reporting new human mutations in a patient cohort and analysing the phenotype of the corresponding yeast strains are still very rare (Baruffini et al. 2011). Furthermore, for all the modelled mutations, we observed phenotypes consistent with the clinical status of the human patients.

All the mutant strains in our yeast model display a phenotype that in some aspects differs significantly from the wild type. Arg770Glu and Val863Ala, corresponding to human p.Gln968Glu and p.Val1106Ala *POLG* variants, respectively, are located in the polymerase domain and show a rather mild defect of mtDNA stability (measured by a moderate increase in *petite* frequency). Additionally, in both these strains, we observed an increase in the frequency of point mutations (measured by the rates of spontaneous erythromycin and oligomycin resistant colony formation). This correlates with the observations made in patients—proband EII2 carrying the p.Val1106Ala variant has no detectable mtDNA lesions, in spite of polyneuropathy symptoms, whereas in the muscle tissue of proband CII1 carrying the p.Gln968Glu variant, multiple DNA deletions are evident, but the phenotype is still relatively mild (PEO). Correspondingly, the decrease in mtDNA stability and increase in the frequency of point mutations were more pronounced in the yeast strain carrying the Arg770Glu variant than in the one with the Val863Ala allele. The relevance of the relatively mild increase in the frequency of point mutations we observed for these variants in the yeast model for the human pathology is, however, not evident. In yeast, a mutant deficient in the exonuclease function of Mip1p (Exo^−^) shows an increase in mtDNA point mutations of about 550-fold (Stumpf et al. 2010) that is orders of magnitude larger than the effect of the Arg770Glu and Val863Ala alleles (about 15-fold at best). Also, increases in mtDNA mutation rate in the exonuclease deficient *POLG* mouse model (Trifunovic et al. 2004; Vermulst et al. 2007) lead to a premature ageing phenotype, but do not result in marked physiological effects in young animals. The effects of changes that involve mtDNA point mutation frequencies are likely to be cumulative, and thus difficult to compare between different model systems.

The other two polymerase domain mutations are Arg672Ter and Thr809Ter, corresponding to human variants p.Arg869Ter and p.Thr1053Argfs*6, respectively. These two alleles encode protein variants with a sequence truncated in the middle of the polymerase domain, due to the presence of a premature STOP codon. Expectedly, in the monoallelic yeast strains, we observe a total loss of Mip1 activity, resulting in a complete loss of functional mtDNA. Accordingly, compound heterozygote patients carrying these variants exhibited a severe Alpers syndrome (Table 1). In the yeast model, in the heteroallelic setting (with a mutated allele and the wild-type allele present in the same strain), they show a partially dominant effect, with a significant, albeit not complete, loss of mtDNA stability (Fig. 4b; Table 2) compared to a hemiallelic control. In the case of Thr809Ter, the partially dominant effect shows an allele dosage dependency (Fig. 4c), as increasing the copy number of the mutated allele by expressing it from a multicopy plasmid results in a further increase in *petite* colony formation, suggesting that this mutation is antimorphic.

While it is difficult to speculate about the exact molecular mechanism of the observed defect without in-depth biochemical experiments, there are several possible mechanisms that could explain the disruptive effect of these mutations, resulting in a partially dominant negative phenotype. The truncated proteins could disrupt replication by competing for the binding of substrates or other protein factors essential for replication. Similar dominant negative phenotypes of *mip1* alleles modelling putative human pathological variants were observed in previous studies (Stumpf et al. 2010). Interestingly, in familial analysis of human p.Arg869Ter and p.Thr1053Argfs*6 variants, no mitochondrial disorder symptoms were reported for heterozygous carriers of these alleles. The yeast model system appears to be more sensitive to perturbations in the mtDNA maintenance, as the lack of dominant mitochondrial disease in humans for alleles showing partial dominance in the yeast model was noted for at least five different cases (Stumpf et al. 2010). This could be explained by the lower copy number of mtDNA molecules per cell in yeast (about 20–30 copies per cell, 50–100 fold less than in typical vertebrate cells), and by the generally less robust character of the *S. cerevisiae* mitochondrial genome system, evidenced by the spontaneous generation of petites, evident even in wild-type strains and exacerbated by many different genetic and environmental factors (Contamine and Picard 2000; Lipinski et al. 2010). Alternatively, the carriers may suffer from defects that are very mild compared to the severe pathology in affected compound heterozygotes, and thus are not evident in a typical familial study, or will develop a phenotype later in life. Low penetrance of pathologies such as migraines and neuropathies was suggested for simple heterozygous carriers of strongly pathogenic *POLG* variants (Tzoulis et al. 2006).

Interestingly, the only change in our analysis that is located in the exonuclease domain of the polymerase γ enzyme, p.Arg309Cys, corresponding to Arg265Cys in *S. cerevisiae* Mip1p, results in a yeast phenotype that is as severe as that of the truncated polymerase domain variants described in the previous paragraph, with a complete loss of functional mtDNA in the monoallelic strain and a significant increase in petite frequency in the heteroallelic setting (Table 2; Fig. 4). Such a strong phenotype is unexpected for a simple substitution in the exonuclease domain, but stands in agreement with an original report of a patient with homozygous p.Arg309Cys variant, resulting from parents consanguinity, having way more severe phenotype than patient AI2 who is a compound heterozygote. Unlike proband AI2, the p.Arg309Cys homozygote started showing symptoms during adolescence and besides PEO, ataxia, myopathy and dyshartia she also suffered from chronic intestinal pseudo-obstruction, epilepsy, strokes, hearing loss and pigmentary retinopathy, which led to her death at the age of 22 (Amiot et al. 2009). The phenotype of this variant is probably not related to an essential character of the mutated residue, as a different substitution in the same position; Arg265Leu results only in a slight increase of petite frequency in yeast (Stumpf et al. 2010) and the human p.Arg309Leu allele in compound heterozygotes [patient CII1 in this study and one patient described previously (Lamantea et al. 2002)] causes only a relatively mild PEO phenotype. A previously described *mip1* mutant completely deficient in the exonuclease function (Exo^−^) shows a massive increase in mtDNA point mutations (about 550 fold), but only about 10 % *petite* frequency (Stumpf et al. 2010). The severe phenotype of Arg265Cys could be due to the presence of an additional cysteine residue which can form undesirable disulphide bonds with another residue in the protein, and thus seriously alter the protein conformation (Farnum et al. 2014).

To summarize, the yeast model proved to be a valuable tool for the initial assessment of the phenotypic effect of novel or poorly characterised POLG variants found in human patients. The phenotypes of strains with mutations introduced into orthologous positions in regions conserved between human POLG and *S. cerevisiae* Mip1p are in good agreement with clinical observations, as variants found in patients affected with the most severe forms of mitochondriopathy cause a complete loss of mtDNA stability in the monoallelic setting in yeast. The less severe variants corresponded to a partial loss of mtDNA stability (increase of *petite* formation rate) and/or increase in point mutation frequencies.

A recently developed alternative to orthologous modelling is based on the expression of human *POLG* as the only mtDNA polymerase in yeast cells devoid of the native *MIP1* gene, and is termed “humanized yeast” (Qian et al. 2014). Both approaches have their merits and shortcomings, and should be viewed as complementary rather than exclusive. Orthologous modelling is obviously unavailable when the variants occur in sequence regions that are not conserved between human and yeast polymerase γ sequences, like in case of proband AI2 carrying a novel variant p.Arg290Cys in addition to p.Arg309Cys. Using the human mitochondrial polymerase would overcome this particular limitation, but given the substantial differences in the structure and replication of yeast and human mtDNA, phenotypes observed for the variants of the human enzyme could also be related to changes in its capability to function in a heterologous environment.

Yeast Mip1 exhibits amazing sensitivity when used to model putative pathogenic variants of the orthologous human mtDNA polymerase. In the monoallelic setting, even the slight phenotypic effects manifest themselves as observable changes in the mitochondrial genome stability and fidelity of replication. As phenotypic tests involving very large numbers of individual colonies are feasible and economical in yeast, statistical significance of these observations can be reliably assessed. For the severe defects that cause complete loss of functional mtDNA, heteroallelic strains can be easily constructed, and they provide valuable insights into the effects of these alleles, including dominance/recessiveness relationships that can be crucial for understanding the effects of corresponding human variants.

In conclusion, we proved that the mitochondrial DNA polymerase variants discovered in patients with mitochondrial disease, and modelled in yeast, have an influence on different aspects of the mitochondrial genome maintenance, and are thus likely to be *bona fide* pathogenic mutations. Our study also further confirms the utility of yeast Mip1p as a tool for studying human *POLG* variants.

## Electronic supplementary material

Supplementary material 1 (PDF 50 kb)

Supplementary material 2 (PDF 1949 kb)

Supplementary material 3 (PDF 568 kb)
