# Supplementary material for: Yeast model analysis of novel polymerase gamma variants found in patients with autosomal recessive mitochondrial disease
Source: Hum Genet. 2015 Jun 16;134(9):951–66. doi: 10.1007/s00439-015-1578-x (PMC4529462; doi:10.1007/s00439-015-1578-x)
Supplement: Supplementary file 1 — Supplementary material 1 (PDF 50 kb) [file 439_2015_1578_MOESM1_ESM.pdf]

Sequences of primers used in this study

Name	Sequence 5'-3'
POLG1_1F	TCCACGTCTTCCAGCCAGTA
POLG1_1R	AGCACGTAACAGGACCTCAG
POLG1_2F	AGTGGTTGTTGTGGAGTGGA
POLG1_2R	AACCACTGAGATTAGGGCTC
POLG1_3F	AGGTCCACACCACCAAGCAG
POLG1_3R	CAGCGCCACCTGATTACAGT
POLG1_4F	AGTCTTGCCTCCTGTGGTCA
POLG1_4R	CTGTCCTGAGAATGGAGCAA
POLG1_5F	TGTGAGAGAGAGAACCTTCC
POLG1_5R	TAGCCTGAGCTGACCAGCCA
POLG1_6F	GTGGGCATCTGGTAATCAGC
POLG1_6R	GAGCAGAATGAGGAAACACC
POLG1_7F	GAGTGCAGGTACTCACGTTG
POLG1_7R	AAGAGAGGGGAAAGGCATCC
POLG1_8F	TAGATTCTGCTTCCCATGGC
POLG1_8R	ACAGACCTGGGAGAGGAAGA
POLG1_9F	TCAACTCTGGCTCCAGGAAT
POLG1_9R	TAAAGCAGGCCTCGGGTCCT
POLG1_10F	AGCGTGGCACAGGAAGCACT
POLG1_10R	AGCCCCACATAGGAGCACAT
POLG1_11F	GTGATGCATCTGTTCACAGG
POLG1_11R	TCCACCTCAGATCCTATGTG
POLG1_12F	AGCTCCTTTGCTCACTTCTG
POLG1_12R	ATGGCTGGCCTTAGGCAAGC
HRM869F	CTCACTCTTCCCACAGCCTG
HRM869R	TCCACATCAGCACCCACAAG
HRM968F	TTCAACTACGGCCGCATCTATGGT
HRM968R	CCTTGGTGGCAGCGTACATCTG
H3POLG1_ 3F	AGAAGCTTAGGTCCACACCACCAAGCAG
S1POLG1_ 3R	GTCGACGGCAGCGCCACCTGATTACAGT
MipSlicF	TATGACCATGATTACGCCAAGCTTGCATGCCTGCAGGTCGACAGCCTTGACAATCGCC
MipSlicR	CGACGTTGTAAAACGACGGCCAGTGAATTCGAGCTCACGTTAGTGGCATTATCAGC
Mip1RsF	CGAGGTCGACGGTATCGATAAGCTTAGCCTTGACAATCGCCTTAG
Mip1RsR	CGGCCGCTCTAGAACTAGTGGATCCGAGCTCACGTTAGTGGCATTATCAG
R265Cl	GTGTTCAAGACAATGTCCGATGTTCATGAAG
R265Cr	CATGAACATCGGACATTGTCTTGAACACAG
R672STOPl	TGCAAAAGCAAATTAGATAGGTTCCGAATTGA
R672STOPr	TCGGAACCTATCTAATTTGCTTTTGCATTG
R770El	GCGAGTCAGTTACTTAAAGAGTTTAACCCATCTC

R770Er	GAGATGGGTTAAACTCTTTAAGTAACTGACTCGC
R770Ql	GCGAGTCAGTTACTTAAACAGTTTAACCCATCTC
R770Qr	GAGATGGGTTAAACTGTTTAAGTAACTGACTCGC
del809STOPl	TTTGGTATGGTGGATGAGTCAATTCTATTC
del809STOPr	GAATAGAATTGACTCATCCACCATACCAAAA
V863Al	ATCCAGTCATCTGGAGCGGATTATCTACAT
V863Ar	ATGTAGATAATCCGCTCCAGATGACTGGATG
rtMipL	GCAACGAGGGACAAGTATG
rtMipshR	CTTTACCACCGCAACTACC
rtMiploR	CGTGATGAGCGTCTTCTTC
