# Supplementary material for: Yeast model analysis of novel polymerase gamma variants found in patients with autosomal recessive mitochondrial disease
Source: Hum Genet. 2015 Jun 16;134(9):951–66. doi: 10.1007/s00439-015-1578-x (PMC4529462; doi:10.1007/s00439-015-1578-x)
Supplement: Supplementary file 2 — Supplementary material 2 (PDF 1949 kb) [file 439_2015_1578_MOESM2_ESM.pdf]

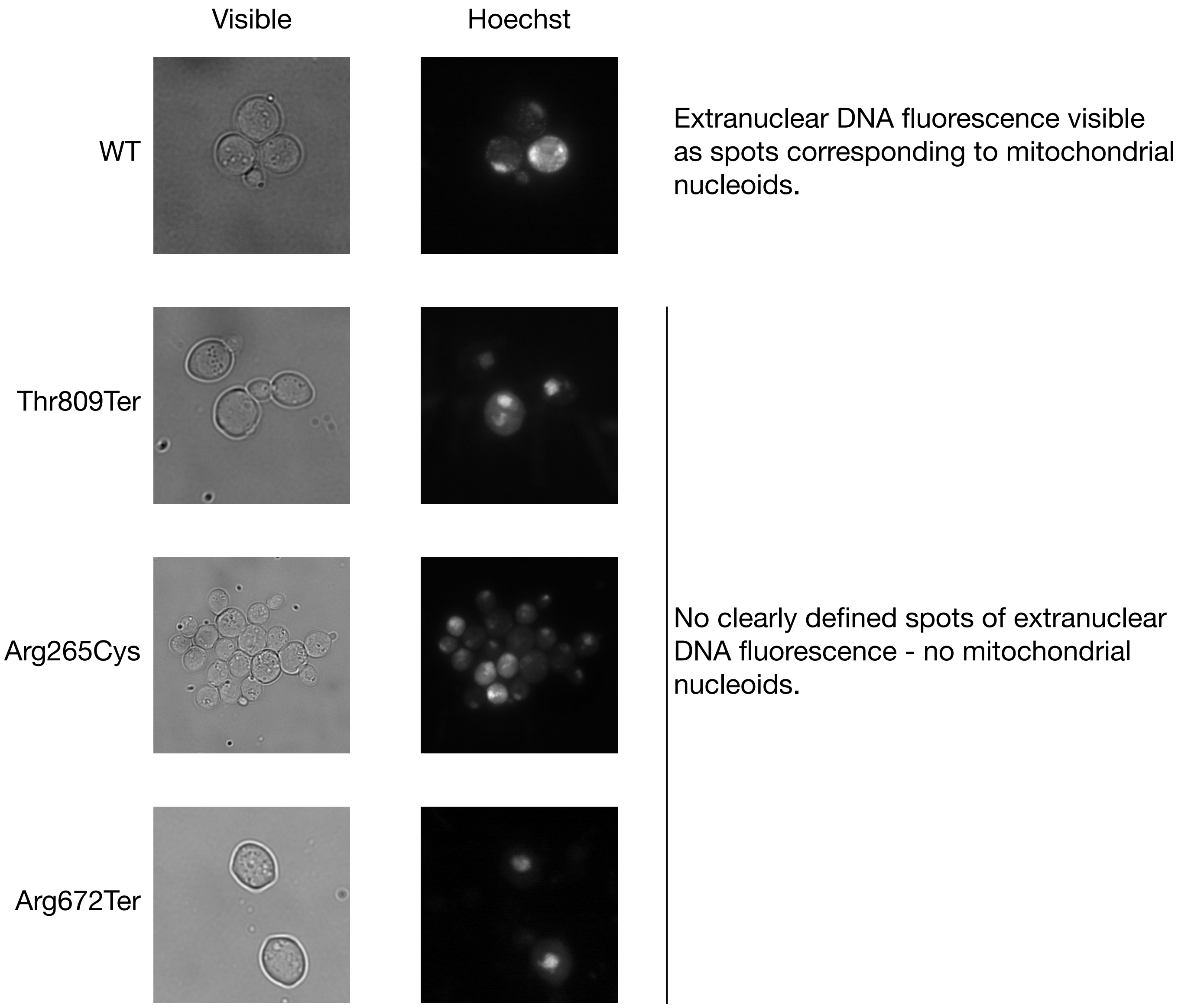

Visible
Hoechst
WT
Thr809Ter
Arg265Cys
Arg672Ter
Extranuclear DNA fluorescence visible as spots corresponding to mitochondrial nucleoids.
No clearly defined spots of extranuclear DNA fluorescence - no mitochondrial nucleoids.
