# Supplementary material for: Yeast model analysis of novel polymerase gamma variants found in patients with autosomal recessive mitochondrial disease
Source: Hum Genet. 2015 Jun 16;134(9):951–66. doi: 10.1007/s00439-015-1578-x (PMC4529462; doi:10.1007/s00439-015-1578-x)
Supplement: Supplementary file 3 — Supplementary material 3 (PDF 568 kb) [file 439_2015_1578_MOESM3_ESM.pdf]

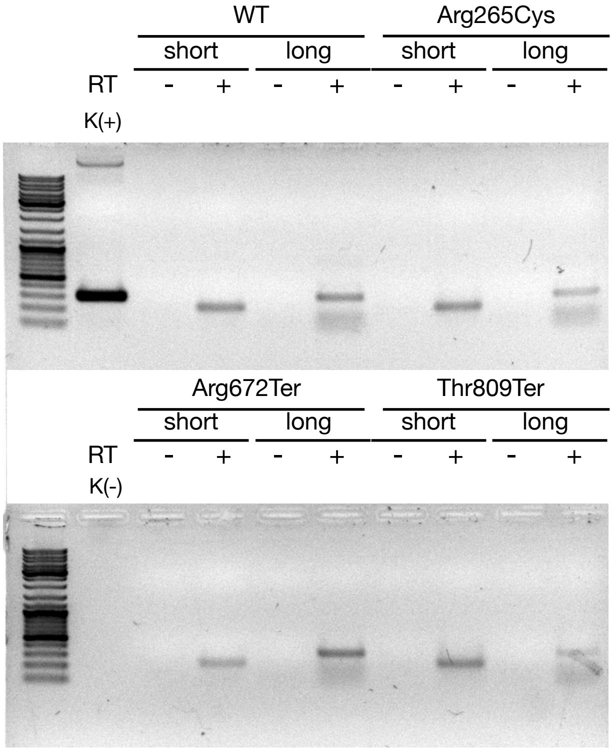

RT-PCR analysis of *MIP1* expression in strains carrying deleterious variants as the only allele (homoalllelic). RT + or - refers to reactions with reverse transcriptase (RT) and controls without RT, respectively. “Short” and “long” refer to two pairs of RT-PCR primers (see Materials and methods). K(+) is the positive control, with a wild type *MIP1* cloned in a plasmid used for PCR, K(-) is the negative PCR control (no template).
